# Emerging and Established Histological Techniques for the Analysis of Thrombosis in COVID-19 Lungs

**DOI:** 10.3389/fcvm.2021.745906

**Published:** 2021-09-21

**Authors:** Addie B. Spier, Colin E. Evans

**Affiliations:** ^1^Department of Medicine, University of Illinois College of Medicine, Rockford, IL, United States; ^2^Department of Pediatrics, Lung and Vascular Biology Program, Stanley Manne Children's Research Institute, Ann & Robert H. Lurie Children's Hospital of Chicago, Chicago, IL, United States; ^3^Department of Pediatrics, Division of Critical Care, Northwestern University Feinberg School of Medicine, Chicago, IL, United States

**Keywords:** thrombosis, lung, histology, pulmonary, COVID - 19

## Abstract

Coronavirus disease 2019 (COVID-19) is the potentially lethal disease that is caused by severe acute respiratory syndrome coronavirus 2 (SARS-CoV-2). Patients with COVID-19 have an increased risk of thrombosis, but the role of thrombosis in the pathogenesis and progression of severe COVID-19 remains unclear. A better understanding of the contribution of thrombosis to the development and progression of COVID-19 could lead to the development of novel COVID-19 treatments. For this reason, established and emerging histological techniques have recently been used to analyze COVID-19 lungs quantitatively and visually and in two and three dimensions. The gold standard and novel state-of the-art histological techniques that have been used to assess thrombosis in COVID-19 lungs are described in this Mini Review.

## Background

Coronavirus disease 2019 (COVID-19) is the highly contagious and potentially debilitating disease caused by severe acute respiratory syndrome coronavirus 2 (SARS-CoV-2), which is associated with increased levels of pulmonary thrombosis (1–3). The COVID-19 pandemic has resulted in massive global suffering (4) including job losses, travel restrictions (5), mental and physical illness (6, 7), and mortality (7). Several treatments for COVID-19 have been given emergency use authorization by the FDA, but these treatments are less than optimal. Remdesivir, for example, improves time to recovery in hospitalized patients with moderate disease (NIAID ACTT-1 trial), but has not been shown to improve survival (8, 9). A major limitation of Remdesivir is its requirement for intra-venous delivery. Another example, Dexamethasone, reduces mortality in persons with severe COVID-19 (RECOVERY trial) (10), but corticosteroid treatment in patients with SARS-CoV, MERS, and influenza can result in increased mortality, incidence of secondary infections, and impaired viral clearance. Convalescent plasma is another treatment granted emergency use authorization by the FDA for hospitalized COVID-19 patients, but a mortality benefit of this treatment has not yet been shown (11). The FDA has also granted emergency use authorization to monoclonal antibodies, for the treatment of non-hospitalized mild to moderate COVID-19. These treatments have importantly shown a reduction in hospitalization in those at high risk for disease progression (BLAZE-1 trial) (12, 13). However, monoclonal antibodies are not approved for hospitalized or critically ill individuals requiring mechanical ventilation The REMAP-CAP and RECOVERY trials are also assessing the application of Tocilizumab and Sarilumab, which are interleukin 6 receptor antagonists, in critically ill COVID-19 patients (14). Another immunomodulator, Baricitinib, which inhibits januse kinase 1 and 2, has been shown to improve survival in COVID-19 patients receiving Baricitinib plus Remdesivir versus Remdesivir alone (15). Regarding the use of Ivermectin, current evidence is inconclusive (16, 17), but additional randomized controlled trials are underway.

While clinical studies of COVID-19 treatments crucial are ongoing, including tests of anti-coagulant therapies (18, 19), an improved understanding of the pathogenesis and pathological characteristics of COVID-19 may lead to the development of novel treatment options. To this end, several studies have used established or emerging histological techniques to characterize the pathological features of lung injury in COVID-19 patients, including widespread pulmonary thrombosis. Such techniques can also be useful for disease diagnosis or retrospective tissue analysis. For example, a recent study has demonstrated the potential for high-resolution cleared-tissue microscopy in the 3-dimensional (3D) analysis of thrombosis in COVID-19 lungs (20). Although this proof-of-concept study was limited to a single patient, such emerging technological advances enable for the analysis of thrombosis in COVID-19 lungs in cubic millimeter volumes and provide novel insights into COVID-19 disease pathogenesis. While the organ-specific pathological characteristics of COVID-19 have been reviewed elsewhere (21–26), this mini-review describes the 2D and 3D histological studies of COVID-19 lungs, with a focus on thrombosis and vascular abnormalities. To this end, we searched PubMed and Google Scholar for articles that included the following terms: “coagulation or thrombosis or thrombus” and “COVID-19 or COVID19” and “histological or histology” and “lung or pulmonary”.

## Two-Dimensional Histology of COVID-19 Lungs

### Features of Coagulopathy

The first open autopsy histological analysis of dissected lung used hematoxylin and eosin (H&E) staining, fluorescence/immunostaining, and electron microscopy in a thorough 2-dimensional (2D) analyses of 10 COVID-19 patients (27). In their study, Fox et al. showed pulmonary thrombotic microangiopathy including platelet aggregation, platelet-rich microthrombi formation, fibrin deposition, and hemorrhage, with evidence of the activation of megakaryocytes contributing to small vessel clot formation (27). The pulmonary megakaryocytes exhibited nuclear hyperchromasia and atypia, were located in alveolar capillaries, and were found to be producing platelets (27). CD4^+^ lymphocytes were also found to aggregate around small blood vessels, some of which appeared to contain platelets and microthrombi (27). Notably, the presence of microthrombi was specific to the lung tissue and the SARS-CoV-2 pandemic (27). In another study of 6 postmortem lung samples from COVID-19 patients, Eckermann et al. showed conventional histopathological evidence of microthrombi formation and fibrin deposition (28). Microthrombi formation, fibrin deposition, and hemorrhage were also shown in another histopathological study of 31 deceased COVID-19 patients (29). Ackermann et al. examined the lungs of 7 patients who died from COVID-19 by seven-color immunohistochemical analysis, micro–computed tomographic imaging, scanning electron microscopy, and corrosion casting (30). In their study, pulmonary microthrombi formation and fibrin deposition was found (30). In a post-mortem examination study of 7 COVID-19 patients, Rapkiewicz et al. employed H&E staining, immunostaining, and electron microscopy to demonstrate evidence of platelet-rich thrombi in the pulmonary microvasculature and thrombus formation in large pulmonary arteries (31). By studying H&E-stained lung sections from 8 autopsy cases from patients with COVID-19, Kianzad et al. also found histological evidence of pulmonary thrombosis (32). Similarly, Romanova et al. (33), Bryce et al. (34), Bidari Zerehpoosh et al. (35), Mauad et al. (36), Bruce-Brand (37), Elsoukkary et al. (38), and Grosse et al. (39) used H&E staining to show evidence of lung thrombosis in COVID-19 autopsy samples. H&E staining and immunofluorescence were also employed by Nicolai et al. (40) and Oprinca et al. (41) in their demonstrations of pulmonary thrombosis in COVID-19 lungs. A combination of H&E staining, immunostaining, and electron microscopy were used by Falasca et al. (42) and Carsana et al. (43), to show pulmonary microthrombi, fibrin deposition, and hemorrhage in COVID-19 patients at postmortem. Microthrombi containing neutrophil extracellular traps were also shown using immunofluorescence staining, by Middleton et al., in 3 COVID-19 lung autopsies (44).

### Features of Vasculopathy

An early case report of post-mortem lung biopsy using 2D H&E staining revealed diffuse alveolar damage, hyaline membrane formation, interstitial lymphocyte infiltration, and multinucleated syncytial cells in alveolar spaces showing viral cytopathic-like changes (45). Diffuse alveolar damage, hyaline membrane formation, and edema was also found in 2D analyses of dissected lung from open autopsy COVID-19 patients (27). These lungs showed inflammatory cell infiltration (i.e., CD4^+^ and CD8^+^ lymphocytes) in the interstitial spaces and adjacent to large bronchioles and blood vessels (27). Furthermore, RNA imaging and electron microscopy demonstrated fused pneumocytes within alveolar spaces, which contained substantial amounts of RNA and may represent virally infected cells (27). Using H&E staining and scanning electron microscopy, Varga et al. assessed the lung tissue of 3 COVID-19 patients at post-mortem (46). Histological analyses revealed inflammatory cell accumulation in association with the endothelium, which exhibited evidence of viral elements (46). Evidence of endothelial cell apoptotic bodies, small lung vessel congestion, and septal thickening was also found (46). Diffuse alveolar damage and hyaline membrane formation, as well as evidence of emphysema, was also found in 2D slices of COVID-19 lungs by Eckermann et al. (28). Meanwhile, Sadegh Beigee et al. (29) found evidence of the following microscopic features in a cohort of 31 deceased COVID-19 patients: hyaline membrane formation; interstitial leukocyte infiltration; and edema. Ackermann et al. (30) also found evidence of the following features in a cohort of 7 COVID-19 post-mortem lung samples: diffuse alveolar damage; edema; angiotensin-converting enzyme 2 (ACE2) expression in epithelial and endothelial cells; endothelial damage and viral infection; and T-cell infiltration. In an autopsy study by Rapkiewicz et al., lungs showed diffuse alveolar damage with hyaline membranes, pneumocyte hyperplasia, perivascular lymphocyte infiltration, and viral inclusions in macrophages and epithelial cells (31). More recently, Qin et al. used H&E staining and immunofluorescence to show histological evidence of endothelial dysfunction (in the form of VCAM1 staining) in an autopsied lung collected from a severe COVID-19 patient, as well as extensive edema, and evidence of viral staining (47). In H&E-stained lung sections from autopsy cases from COVID-19 patients, Kianzad et al. also found histological evidence of edema, neutrophil infiltration, and diffuse alveolar damage (32). Similarly, Romanova et al. (33), Bryce et al. (34), Bidari Zerehpoosh et al. (35), Mauad et al. (36), Bruce-Brand (37), and Elsoukkary et al. (38) used H&E staining to demonstrate diffuse alveolar damage and leukocyte infiltration in COVID-19 autopsy samples. H&E staining and immunofluorescence were also employed by Nicolai et al. (40) and Radermecker et al. (48) in their demonstrations of neutrophil infiltration and neutrophil extracellular trap formation in COVID-19 lungs. Oprinca et al. used H&E and immunohistochemical staining to show hyaline membrane formation and diffuse alveolar damage in 3 COVID-19 autopsy samples (41). A combination of H&E staining, immunostaining, and electron microscopy were used by Falasca et al. (42) and Carsana et al. (43) in COVID-19 patients at postmortem, to show pulmonary hyaline membranes, inflammatory cell infiltration, diffuse alveolar damage, and vasculitis.

## Three-Dimensional Histology of COVID-19 Lungs

### Features of Coagulopathy

In a study by Li et al., 3D images of lung autopsy tissues were rendered from a single COVID-19 patient (20). This study represents the first report of 3D microscopy images from optically cleared lung tissues from a COVID-19 patient autopsy (20). These authors showed extensive evidence of platelet-rich clotting with adherent mononuclear cells in branching vessels and extensive fibrin clotting in small capillaries (20). The 3D technique used by Li et al. was also able to confirm the finding of activated mature megakaryocytes in small lung vessels, as shown by large, multiple, lobular nuclei (20). Eckermann et al. used multi-scale phase contrast x-ray tomography to analyze postmortem lung samples from 6 COVID-19 patients in 3D (28). In their study, autopsy samples of up to 8 mm thickness were scanned and reconstructed at a resolution that enabled the segmentation of individual cells (28). These studies showed evidence of microthrombi formation and fibrin deposition (28). Ackermann et al. also showed evidence of vessel occlusion by 3D micro computed tomography (30).

### Features of Vasculopathy

In the study by Eckermann et al., 3D virtual histology was used to visualize diffuse alveolar damage, emphysema, hyaline membrane formation, septal thickening, and leukocyte infiltration (28). Li et al. (20) used 3D virtual histology to observe multi-nucleated cells with evidence of viral cytopathic changes, scattered hyaline-fibrin aggregates and inflammatory cells in the alveolar spaces, and diffuse alveolar damage. In the study by Ackermann et al., scanning electron microscopy and microvascular corrosion casting were used to demonstrate structurally deformed capillaries and aberrant angiogenesis in COVID-19 lungs (30).

## Methodological Considerations and Future Perspectives

While both 2D and 3D histological techniques remain temporally limited by the analysis of one timepoint and spatially limited by the amount of tissue that can be imaged, such techniques can provide valuable insights into COVID-19 disease pathology. Often, 2D techniques are quicker and cheaper to perform compared with 3D techniques, but the 3D techniques yield more information and provide improved visualization of disease features. In the manuscript by Li et al. (20), for instance, 3D renderings from a 7.8 mm × 5.9 mm × 0.9 mm formalin-fixed block of lung tissue from the left peripheral upper lobe were generated, which enabled the visualization of lung features from millimeter dimension vessels to single cell nuclei. However, 3D histology from serial sectioning and whole slide scanning with image reconstruction is expensive and time consuming. In future, it is likely that established 2D histological techniques will be complemented with emerging 3D virtual histology. While the histological studies of COVID-19 patients are often limited in sample size, the key findings from studies of COVID-19 lung that have used 2D and 3D techniques appear to be congruent so far (Table 1). However, it remains to be seen what (if any) additional information will be uniquely generated from each type of histological technique. Furthermore, it is important to note that the findings from histological studies are observational and are usually limited to the demonstration of associations between pathological features and death in diseased patients.

**Table 1:** Pathological features of COVID-19 lungs.

**COVID-19 feature**	**2D Histology**	**3D Histology**
Apoptosis	(46)	
Diffuse alveolar damage	(27, 28, 30–39, 41–43, 45)	(20, 28)
Aberrant capillarization	(30)	(30)
Edema	(27, 29, 30, 32, 43, 47)	
Emphysema	(28)	
Hemorrhage	(27, 29, 42)	
Hyaline membrane	(27–29, 31, 41–43, 45)	(20, 28)
Interstitial leukocytes	(27–32, 35, 37, 40, 42, 43, 45, 46)	(20, 28)
Microthrombi	(27–44)	(20, 28, 30)
Neutrophil extracellular traps	(40, 44, 48)	
Septal thickening	(28, 46)	(28)
Viral infection-like changes	(27, 28, 30, 31, 45–47)	(20)

Analysis of 2D lung tissue sections remains the gold-standard for histological examination, but 2D tissue sectioning can create cutting artifacts that confuse interpretation. Pathological interpretation may be improved in future studies by 3D analysis of tissue microstructure (49). Emerging histological techniques have used fluorescence contrast to simulate H&E staining (50, 51) and optical tissue clearing and high throughput sectioning microscopes to acquire 3D virtual histology of different tissue types (49, 52–54). Such 3D virtual imaging techniques could be improved by: (i) increasing resolution with higher aperture waveguide optics, enhanced pixel detector technology, and improvements in holographic reconstruction; (ii) including more than two zoom levels; and (iii) developing cell-specific markers coupled to radiocontrast agents (28). In both 2D and 3D histology, the consistency and reliability of novel and emerging histological techniques should be an area of focus in future studies. It would also be beneficial if 2D and 3D histological techniques could be upscaled and carried out in multiple different patient sub-groups and at various stages of disease severity. Furthermore, it could be useful if histological imaging studies in future could be performed in an automated manner, perhaps eventually in living patients. Methodological factors pertaining to both 2D and 3D histology should be carefully considered when designing and carrying out histological studies of COVID-19 lungs (55, 56). Nevertheless, histological studies will likely continue to improve understanding of COVID-19 pathogeneses and progression, which will add to understanding of COVID-19 from other pre-clinical and clinical observations, and hopefully lead to the development of novel therapies and treatment strategies.

## Conclusions

Novel treatments for COVID-19 patients and an improved understanding of COVID-19 pathogenesis may arise from in-depth analyses of the histological features of COVID-19 lungs, which include enhanced levels of thrombosis. Such analyses will likely be facilitated by novel histological techniques for high-resolution and large-volume imaging of lung structure and pulmonary thrombosis at the microvascular level.

## Author Contributions

CE and AS wrote and edited the manuscript. All authors contributed to the article and approved the submitted version.

## Funding

CE is supported in part by an American Heart Association Career Development Award (19CDA34500000).

## Conflict of Interest

The authors declare that the research was conducted in the absence of any commercial or financial relationships that could be construed as a potential conflict of interest.

## Publisher's Note

All claims expressed in this article are solely those of the authors and do not necessarily represent those of their affiliated organizations, or those of the publisher, the editors and the reviewers. Any product that may be evaluated in this article, or claim that may be made by its manufacturer, is not guaranteed or endorsed by the publisher.

## Abbreviations

2D: 2-dimensional
3D: 3-dimensional
COVID-19: coronavirus disease 2019.
